# Elder and booster vaccination associates with decreased risk of serious clinical outcomes in comparison of Omicron and Delta variant: A meta-analysis of SARS-CoV-2 infection

**DOI:** 10.3389/fmicb.2023.1051104

**Published:** 2023-04-14

**Authors:** Yanhua Wu, Yuchen Pan, Kaisheng Su, Yangyu Zhang, Zhifang Jia, Jiaxin Yi, Haiyong Lv, Lihuan Zhang, Mingyang Xue, Donghui Cao, Jing Jiang

**Affiliations:** ^1^Center of Infectious Diseases and Pathogen Biology, The First Hospital of Jilin University, Changchun, China; ^2^Department of Clinical Epidemiology, The First Hospital of Jilin University, Changchun, China; ^3^The Second Hospital of Jilin University, Changchun, China; ^4^School of Public Health, Jilin University, Changchun, China

**Keywords:** SARS-CoV-2, Omicron, Delta, clinical outcome, meta-analysis

## Abstract

**Background:**

The COVID-19 pandemic brings great pressure to the public health systems. This meta-analysis aimed to compare the clinical outcomes among different virus variants, to clarify their impact on medical resources and to provide evidence for the formulation of epidemic prevention policies.

**Methods:**

A systematic literature search was performed in the PubMed, Embase, and Cochrane Library databases using the key words “Omicron” and “Delta.” The adjusted Risk ratios (RRs), Odds ratios (ORs) and Hazard ratios (HRs) were extracted, and RRs and Rate difference % (RD%) were used to interpret the risk estimates of the outcomes ultimately.

**Results:**

Forty-three studies were included, with 3,812,681 and 14,926,841 individuals infected with SARS-CoV-2 Delta and Omicron variant, respectively. The relative risks of hospitalization, death, ICU admission, and mechanical ventilation use after infection with the Omicron variant were all significantly reduced compared those after infection with the Delta variant (RR_hospitalization_ = 0.45, 95%CI: 0.40–0.52; RR_death_ = 0.37, 95%CI: 0.30–0.45; RR_ICU_ = 0.35, 95%CI: 0.29–0.42; RR_mechanical ventilation_ = 0.33, 95%CI: 0.25–0.44). The change of both absolute and relative risks for hospitalization was more evident (RR = 0.47, 95%CI: 0.42–0.53；RD% =10.61, 95%CI: 8.64–12.59) and a significant increase was observed for the absolute differences in death in the elderly (RD% = 5.60, 95CI%: 4.65–6.55); the change of the absolute differences in the risk of hospitalization and death were most markedly observed in the patients with booster vaccination (RD%_hospitalization_ = 8.60, 95CI%: 5.95–11.24; RD%_death_ = 3.70, 95CI%: 0.34–7.06).

**Conclusion:**

The ability of the Omicron variant to cause severe clinical events has decreased significantly, as compared with the Delta variant, but vulnerable populations still need to be vigilant. There was no interaction between the vaccination doses and different variants.

## 1. Introduction

Coronavirus disease (COVID-19) as an acute respiratory infectious disease was confirmed by the World Health Organization (WHO) on February 11, 2020, which has caused a global pandemic and brought a huge burden to the world’s public health system (Ahn et al., 2020). A significant feature of severe acute respiratory syndrome coronavirus 2 (SARS-CoV-2), which is the causative agent of COVID-19, an RNA virus, is that it can constantly mutate with human transmission (Ciotti et al., 2022). The Delta variant was first reported in India in October 2020 and caused a new wave of global pandemic (European Centre for Disease Prevention and Control, 2021). Compared with the original Alpha variant, the pulmonary infectivity of the Delta variant increased by 51 times, and more likely to caused severe illness (Andrews et al., 2022). Subsequently, on November 25, 2021, WHO confirmed the Omicron variant (B1.1.529; World Health Organization, 2021a), which quickly became the main epidemic variant worldwide with a more dreadful transmission power (Araf et al., 2022). Although the infection rate of the Omicron variant in South Africa, the United States (US), and Europe have increased sharply compared with the Delta variant, but the hospitalization and death rates caused by Omicron variant infection were significantly lower than that of the previous SARS-CoV-2 variants (Lewnard et al., 2022; Nyberg et al., 2022).

Based on the enormous pressure brought by the COVID-19 epidemic to the global public health system, understanding the difference in the clinical outcomes between the current epidemic variant Omicron and Delta is important to formulate more accurate epidemic prevention policies. The multi-level meta-data covering multiple regions worldwide remains limited. Concurrently, the estimation of the absolute risk change is of more significance to public health. Therefore, using data from cohort and registration studies comparing Delta and Omicron variants worldwide, we conducted a meta-analysis to investigate the difference in hospitalization rate and risk of severe clinical events between the Omicron and Delta variants to contribute to the establishment of further public health policies.

## 2. Methods

This analysis was performed in accordance with Preferred Reporting Items for Systematic Reviews and Meta Analyses (PRISMA) guidelines (Page et al., 2021) and registered in PROSPERO.

**Figure 1:**
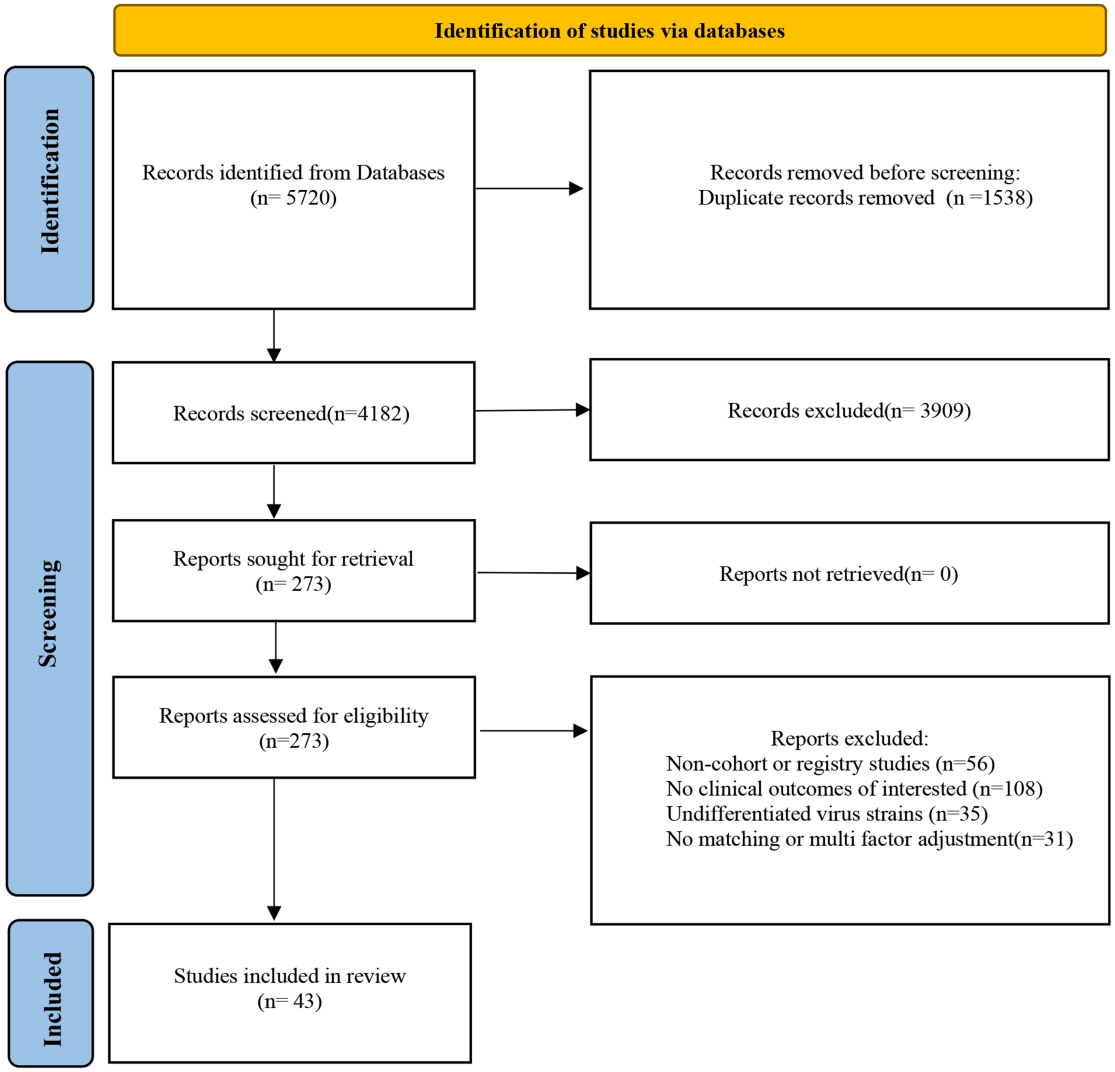
Preferred Reporting Items for Systematic Reviews and Meta Analyses (PRISMA) 2020 flow diagram of study selection and data extraction.

### 2.1. Search strategy

A comprehensive search was conducted on PubMed, Embase, and the Cochrane Library for all relevant articles published from the emergence of COVID-19 on December 24, 2021, to the December 31, 2022. The search terms used were “Omicron” and “Delta.” The reference lists of identified studies and reviews were hand searched for potentially relevant studies not previously identified in the database search.

### 2.2. Article selection

The duplicates were removed. Then, the studies were screened by title and abstract, later followed by full text reviewed by six investigators. In case of disagreement, a consensus was reached by discussion. Studies were included if they fulfilled the following criteria: (1) cohort or registry study; (2) used pre-matched or post-hoc multivariate adjustment; (3) provided at least one of the following clinical outcomes of Omicron and Delta patients: hospitalization, ICU admission, mechanical ventilation use, and death; and (4) the sample size of the study was >100. The exclusion criteria were (1) systematic review, case report/series, editorial, letter, abstract, and animal study; and (2) with overlapping population.

### 2.3. Data extraction and quality assessment

Data, including name of first author, published time, study region, study design, sample size, age, match or multivariate analysis method and variables, and outcome indicator, were extracted using a standardized data collection form. Two researchers independently assessed the study quality using the Newcastle-Ottawa Scale (NOS; Margulis et al., 2014) based on three domains: selection, comparability, and exposure. The highest score is 9 points and studies with scores ≥7 were considered of high quality.

### 2.4. Statistical analysis

Stata 12.0 software (StataCorp, College Station, TX, United States) was used to perform the meta-analysis. The adjusted RRs, ORs, and HRs were used to pool the risk estimates of outcomes when available. When the effects with 95% confidence interval (CIs) were not shown directly in a publication, they were calculated by using a two-by-two frequency table. RRs (Omicron vs. Delta) were used to interpret the risk estimates of outcomes ultimately. The weighted natural logarithm of the RRs with their 95% CIs was used to obtain the pooled relative risk estimates. Statistical heterogeneity was assessed using the I^2^ statistics. If the heterogeneity among studies was >50%, the random-effect model was used. Otherwise, a fix-effect model was chosen. Based on the included articles, we performed subgroup meta-analyses according to the age (Younger group: <=16 years old, or 19 years old, or 20 years old; Medium group: 18 years old to 60/65 years old; Elder groups: >60 years old or 65 years old), vaccination status (Unvaccinated or Insufficient vaccinated, Primary vaccinated and Booster), patient source (general population and others). The Begg’s test was used to evaluate potential publication bias (significant when *p* < 0.05). Sensitivity analyses were performed to evaluate the robustness of the results by omitting studies one by one.

## 3. Results

### 3.1. Characteristics of the included studies

Of the 5,720 articles identified, 273 were eligible for full-text screening; 36 cohort and 7 registry studies (Adjei et al., 2022; Auvigne et al., 2022; Bager et al., 2022; Beraud et al., 2022; Bonsignore et al., 2022; Bouzid et al., 2022; Butt et al., 2022a,b,c; Català et al., 2022; Chanda et al., 2022; Davies et al., 2022; DeSilva et al., 2022; Fall et al., 2022; Goga et al., 2022; Jassat et al., 2022; Kahn et al., 2022; Krutikov et al., 2022; Lewnard et al., 2022; Mayr et al., 2022; Menni et al., 2022; Nevejan et al., 2022; Nyberg et al., 2022; Pascall et al., 2022; Sacco et al., 2022; Shi et al., 2022; Sievers et al., 2022; Skarbinski et al., 2022; Stålcrantz et al., 2022; Stepanova et al., 2022; Strasser et al., 2022; Ulloa et al., 2022; Van Goethem et al., 2022; Vieillard-Baron et al., 2022; Wang L. et al., 2022; Wang X. et al., 2022; Whittaker et al., 2022; Wolter et al., 2022; Wrenn et al., 2022; Esper et al., 2023; Greene et al., 2023; Intawong et al., 2023; Trobajo-Sanmartín et al., 2023) were included finally (Figure 1). Altogether, 3,812,681 and 14,926,841 individuals were infected with the Delta and Omicron variants, respectively. All eligible articles were published in English. The quality scores evaluated by NOS ranged from 7 to 9. The quality of the included studies was high (Supplementary Table S1). Twenty-eight articles were analyzed for clinical outcome of hospitalization; 35 for death; 29 for ICU admission; and 23 for mechanical ventilation (Table 1; Supplementary Tables S2-S5).

**Table 1:** Characteristics of eligible studies.

No	Author	Published month	Region	Study design	Population	Ages	Sample size	Match or multivariate analysis method	Matching factors or adjust variables	Effect index	Reported index			
											Hospitalization	Death	ICU	Mechanical
1	Wang L^a^	2022.02	United States	Retrospective cohort study	General population	All	D: 147,107	1:1 propensity-score matching	Race, ethnicity and gender stratified cohorts, other demographics, socioeconomic factors, COVID-19-related health conditions, medications, and documented vaccination status.	RR	YES	NO	YES	YES
							O: 147,107							
2	Kahn	2022.03	Sweden	Cohort Study	General population	All	D: 13,711	Logistic regression model	Age, sex, comorbidities, prior infection, time since last dose and booster dose.	RR	NO	NO	YES^b^	NO
							O: 29,539							
3	Robert Whittaker^a^	2022.03	Norway	Cohort Study	General children	<18	D: 42,362	Multivariable log-binomial regression model	Variant wave, age, sex, country of birth, region of residence, and underlying comorbidities	RR	YES	NO	NO	NO
							O: 82,907							
4	Pascall^a^	2022.03	UK	Cohort Study	General population	≥18	D: 1,164	cumulative generalized additive mixed models with logit links fit using Bayesian inference	lineage, reinfection, sex, number of vaccine doses, number of ISARIC4C identified comorbidities, age and date of positive test	RR	YES	NO	NO	NO
							O: 2,694							
5	Wang X^a^	2022.03	United States	Cohort Study	General population	All	D: 27,001	Logistic regression model	Vaccination status, prior infection, age, sex, race/ethnicity, smoking status, comorbidities, week of testing, and geographic location	OR	NO	YES^c^	NO	NO
							O: 45,223							
6	Ulloa	2022.04	Canada	Retrospective populationwide matched cohort study	General population	All	D: 9,087	1:1 Matched. Cox proportional hazards regression model	For match: sex, age in years, vaccination status, time since most recent vaccine dose, region, and onset date； For regression: sex, age group, and vaccination status	HR	YES^d^	YES	YES^e^	NO
							O: 9,087							
7	Butt	2022.04	Qatar	Cohort Study	General population	< 18	D: 985	1:1 propensity-score matching. Logistic regression model	For match: age, sex, nationality, and presence of co-morbidities For regression: /	OR	YES	YES^f^	YES^f^	YES^f^
							O: 985							
8	Wrenn	2022.04	United States	Cohort study	General population	All	D: 489	Logistic regression model	Viral variant, age, sex, race, ethnicity, obesity, and SARS-CoV-2 vaccination status	OR	YES	YES	NO	YES
							O: 263							
9	Menni	2022.04	UK	Prospective Cohort Study	General population, at least 2 doses of vaccine	≥ 16	D: 4,990	1:1 using Euclidean distance-based algorithm matching. Logistic regression model	For match: age, sex, vaccination doses. For regression: age, sex, and vaccination doses	OR	YES	NO	NO	NO
							O: 4,990							
10	Nyberg	2022.04	UK	Retrospective cohort study	General population	All	D: 448,843	Cox proportional hazards regression model	Sex, index of multiple deprivation, year of age within each age band, and an interaction term between previous infection status and any history of vaccination	HR	YES	YES	NO	NO
							O: 1,067,859							
11	Shi	2022.04	United States	Registry study	Hospitalized children	5 to 11	D: 482	Multivariable generalized estimating equations	Demographic characteristics, underlying medical conditions, and variant periods	RR	NO	YES	YES	YES
							O: 397							
12	Krutikov	2022.05	UK	Cohort Study	Residents of Long-Term Care Facilities	≥ 65	D: 400	Cox proportional hazards regression model	Age, sex, past infection, primary vaccination type, and time from booster vaccination, with exploration for evidence of an interaction with omicron period for all adjustment variables	HR	YES	YES	NO	NO
							O: 1,864							
13	Sacco	2022.05	Italy	Cohort Study	Individuals with at most one episode of reinfection	All	D: 6,030	Negative binomial generalized linear mixed model	Severe SARS-CoV-2 reinfections VOC predominance phase, severity of first SARS-CoV-2 infection, vaccination status, age group, sex, healthcare worker status and nationality.	IRR	YES^d^	YES^d^	NO	NO
							O: 163,468							
14	Vieillard	2022.05	France	Registry study	Hospitalized patients	All	D: 400	Cox proportional hazards regression model	Age, time from symptoms onset to ICU admission, vaccination status and immunosuppression	RR	NO	YES	NO	YES
							O: 229							
15	Fall	2022.05	United States	Retrospective cohort study	Inpatients and Outpatients	All	D: 908	Logistic regression model	Age, gender, race and ethnicity, and comorbidities	OR	YES	YES	YES	NO
							O: 1,119							
16	Butt	2022.05	United States	Retrospective cohort study	General population	>20	D:2,619	1:1 matching Cox proportional hazards models	For match: age,sex, race, Charlson Comorbidity Index vaccine type, calendar week of second vaccine dose, and geographic site of second vaccine dose administration. For regression::all variables used for matching	OR	YES	YES^e^	YES^e^	NO
							O:18,906							
17	Lewnard	2022.06	United States	Registry study	General population	All	D: 23,305	Cox proportional hazards regression model	Age; sex; race/ethnicity census; tract-level median household income; smoking status; body mass index; Charlson comorbidity; prior-year emergency department visits and inpatient admissions；documented prior SARS-CoV-2 infection; and history of COVID-19 vaccination	HR	YES	YES	YES	YES
							O: 222,688							
18	Bouzid	2022.06	France	Retrospective Cohort Study	Emergency patient	≥16	D: 818	Logistic regression model	Age, sex, hypertension, obesity, diabetes, chronic respiratory disease, chronic kidney disease, immunosuppression, number of vaccine doses, and center.	AR (Transformed to RR)	YES	YES	YES	YES
							O: 898							
19	Davies	2022.06	South Africa	Cohort Study	General population	≥ 20	D: 4,355	Cox proportional hazards regression model	Age, sex, geographic location, comorbidities, vaccination, and prior diagnosed infection	RR	YES^d^	YES	YES^g^	YES^g^
							O: 5,104							
20	Sievers	2022.06	Germany	Retrospective cohort study	General population	All	D: 24,530	Logistic regression model	Age, vaccination status, sex, federal state of notifying health authority and week of notification	OR	YES	YES	YES	NO
							BA.1: 163,468							
							BA.2: 6,860							
21	Auvigne	2022.06	France	Retrospective Cohort Study	General population	≥18	D: 92,182	Cox proportional hazards regression model	Age, sex, vaccination status, presence of comorbidity and region of residence.	HR	NO	YES^e^	YES^e^	NO
							O: 92,182							
22	Stålcrantz	2022.06	Norway	Cohort Study	Hospitalized patients	All	D: 666	Cox proportional hazards regression model	Sex, age, country of birth, risk factors, regional health authority and vaccination status	HR	NO	YES	YES	NO
							O: 409							
23	Van	2022.06	Belgium	Retrospective cohort study	Hospitalized patients	≥18	D: 509	Matched weighted logistic regression model	For match: hospital For regression: age, gender, comorbidity, place of infection, educational level, income, population density at postal code level, vaccination status at diagnosis, mean ICU occupancy rate during the patients hospital stay, and two-way interactions of these covariates	RR	NO	YES	YES	YES
							O: 445							
24	Skarbinski	2022.06	United States	Retrospective cohort study	General population	All	D: 69,977	Cox proportional hazards regression model	Sex, age, ethnicity, Charlson comorbidities index score and selection comorbidities, BMI, prior infection, receiving anti-SARS-CoV-2 monoclonal antibody therapy, vaccination status	HR	YES	YES	NO	YES
							O: 48,101							
25	Mayr	2022.06	United States	Retrospective Cohort Study	Veterans	≥18	D: 22,841	1:1 Matched. logistic regression model	Gender, age, number of chronic health conditions, vaccination status, week of 2nd vaccination, socioeconomic status, and VA medical center	RR	YES	YES^e^	YES^e^	YES
							O: 22,841							
26	Jassat	2022.07	South Africa	Cohort Study	General population	All	D: 1,306,260	Logistic regression model	Age, sex, race, presence of a comorbidity, type of health sector, and province of hospitalization	RR/OR	YES	YES^h^	YES^h^	YES^h^
							O: 629,617							
27	Bager	2022.07	Denmark	Retrospective cohort study	General population	All	D: 150,311	Poisson regression model	Reinfection status, sex, age, region, comorbidities, and time period.	RR	YES	NO	NO	NO
							O: 38,669							
28	Goga	2022.07	South Africa	Cohort form trial	General population	≥18	D: 15,195	Logistic regression model	Adjusted for age, gender, province, clustering, HIV, hypertension, diabetes, and ward	OR	YES	NO	YES	YES
							O: 26,393							
29	Butt	2022.07	Qatar	Cohort Study	General population	≥18	D: 3,926	1:1 propensity-score matching. Logistic regression model	For match: age, gender, nationality, vaccination status at time of infection, and co-morbidities For regression: Vaccination status, age, sex, nationality, comorbidities count	OR	YES	YES^f^	YES^f^	YES^f^
							O: 3,926							
30	Greene	2022.07	United States	Cohort study	General population	All	D: 158,799	Poisson regression model	Gender, age group congregate setting residence, and for community-dwelling residents, neighborhood poverty level	RR	YES	YES	NO	NO
							O: 488,053							
31	Wolter	2022.07	South Africa	Registry study	General population	All	D: 1,273	Logistic regression models	Age, sex, presence of co-morbidity, province and healthcare sector and factors associated with severity (age, presence of co-morbidity, sex, province, healthcare sector, number of days between the dates of specimen collection and hospital admission and SARS-CoV-2 vaccination status)	RR	YES	YES^g^	YES^g^	YES^g^
							BA.1: 75,763							
							BA.2: 20,068							
							BA.4/BA.5: 1,806							
32	Bonsignore	2022.08	Germany	Registry study	Hospitalized patients	≥18	D: 12,370	Logistic GLMMs	Hospitals	OR	NO	YES	YES	YES
							O: 21,222							
33	Stepanova	2022.08	United States	Retrospective cohort study	Hospitalized patients	≥18	D: 860	Logistic or generalized linear regression model	Age，Sex，BMI，CCI，ECI	RR	NO	YES	YES	YES
							O: 1,556							
34	Català	2022.08	Spain	Cohort study	General population	>10	D: 997,748	Mantel–Haenszel method	Age and vaccination status	RR	YES	NO	YES	NO
							O:11,121,316							
35	Esper	2022.10	United States	Registry study	General population	All	D: 808	Logistic regression	Age, sex, comorbidity, vaccination status, and virus lineage	RR	YES	YES	YES	YES
							O:696							
36	Strasser	2022.10	England	Retrospective cohort study	Hospitalized patients	All	D: 20,770	Matched weighted logistic regression model	Sex, age, race and ethnicity, comorbidities, vaccine status, treatments, and prior infection	RR	YES	YES	YES	YES
							O:28,940							
37	Intawong	2022.11	Thailand	Cohort study	General population	≥18	D: 17,047	Cox proportional hazards regression model	Age, gender, calendar day of test, vaccination status and schedules, and time since last vaccine	HR	NO	YES	NO	YES
							O:188,043							
38	DeSilva	2022.11	United States	Retrospective cohort study	Hospitalized patients	≥18	D:16,078	Multivariable logisticregression，Fine-Gray competing risks models, logistic accelerated failure time models	Age, geographic region, calendar time of index date, and local virus circulation and inverse probability weighted by propensity to be vaccinated or unvaccinated	RR	NO	YES	YES	YES
							O:11,071							
39	Nevejan	2022.12	Belgium	Retrospective cohort study	Hospitalized patients	≥18	D: 187	Mixed-model logistic regression analysis	Age at admission, sex, VOC, immune status at admission, vaccination status and time since last vaccination	OR	NO	YES	YES	NO
							O: 1,036							
40	Beraud	2022.12	Bulgaria, Croatia, France, Turkey	Retrospective cohort study	Hospitalized patients	All	D: 955	Multivariate logistic regression	Gender, Age, Diabetes, HTA, Kidney Failure, O2_home,CardiacFailure, ImmunoSup, previous SARS-CoV-2 infection, Vaccination, PulmDis, SolidCancer 3 M, HematoK, OneComorb.	OR	NO	YES^e^	YES	YES
							O: 1,215							
41	Chanda	2022.12	Zambia	Retrospective cohort study	Hospitalized patients	All	D: 752	Multivariate logistic regression	Age, sex, number of comorbid conditions, disease severity at admission, hospitalization month, COVID-19 treatment center	OR	NO	YES	NO	NO
							O: 901							
42	Adjei	2022.12	United States	Registry study	Hospitalized patients	All	D: 163,094	GEE model, log-linked binomial regression	Age, sex, race and ethnicity, number of underlying medical conditions, and presence or absence of a disability	RR	NO	YES	NO	YES
							Early O:104,395							
							Later O:20,655							
43	Trobajo	2022.12	Navarra, Spain.	Cohort study	General population	All	D: 487	Logistic regression models	Sex, age, immunocompromised status, other major chronic conditions, and vaccination status	OR	YES	YES	YES	NO
							O: 1,867							

a The article is a preprint which has not been peer reviewed.

b Need of oxygen supply ≥ 5 L/min or admittance to an intensive care unit (ICU).

c Admission to an intensive care unit, received oxygen treatment, was ventilated, received extracorporeal membrane oxygenation, had acute respiratory distress syndrome, or had died.

d Hospitalization or death.

e ICU admission or death.

f Mechanical ventilation or ICU admission or death.

g Admission to an intensive care unit, mechanical ventilation, or prescription of oral or intravenous steroids.

h Acute respiratory distress syndrome, receipt of oxygen or invasive mechanical ventilation, treatment in high-care or intensive-care units (ICUs), or death.

### 3.2. Omicron variant could reduce half of the relative risk of hospitalization compared with Delta variant

**Figure 2:**
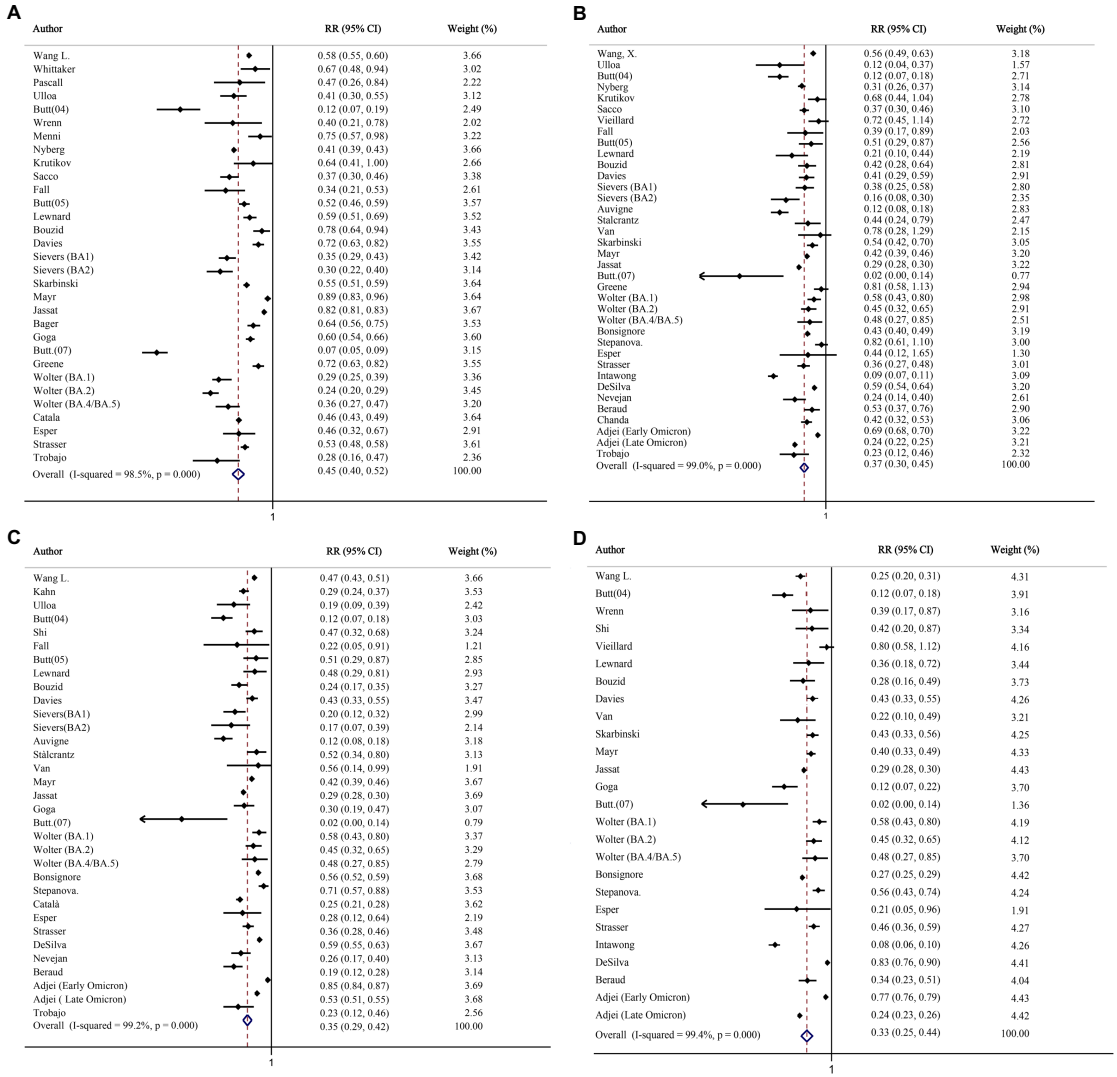
The forest plots of risk of hospitalization, death, intensive care unit (ICU) admission, and mechanical ventilation (Omicron vs. Delta). **(A)** Risk ratio of hospitalization. **(B)** Risk ratio of death. **(C)** Risk ratio of ICU admission. **(D)** Risk ratio of mechanical ventilation.

The analysis of hospitalization included 14,380,294 and 3,446,840 individuals infected with the Omicron and Delta variants, respectively. Heterogeneity was observed among these studies (*I*^2^ = 98.5%; *p* < 0.001); hence, effect size was calculated *via* the random-effects model. The summary RR was 0.45, indicating a statistically significant decreased risk of hospitalization with Omicron variant compared to Delta variant (Figure 2A). Similarly, the same trend was observed in the pooled RD% (4.11, 95%CI: 3.63–4.59; Table 2).

**Table 2:** Pooled RD (Delta-Omicron) for severe clinical outcomes (Delta–Omicron).

	Number of studies	RD (95% CI) per 100 persons
Hospitalization	28	4.11 (3.63–4.59)
Death	35	3.10 (2.67–3.53)
ICU Admission	29	3.05 (2.59–3.51)
Mechanical ventilation	23	4.93 (4.04–5.83)

### 3.3. Omicron variant could reduce the relative risk of severity by two-thirds compared with Delta variant

Altogether, 2,440,093 and 3,473,226 individuals infected with the Delta and Omicron variants, respectively, were included in the analysis of death. The risk of death after the Omicron infection was significantly reduced, as compared with that after the Delta infection (RR = 0.37, 95% CI: 0.30–0.45; RD% = 3.10, 95% CI: 2.67–3.53; Figure 2B; Table 2).

Twenty-night studies (2,884,116 Delta-infected and 12,793,577 Omicron-infected individuals) and Twenty-three studies (1,813,826 Delta-infected and 1,584,424 Omicron-infected individuals) were included in the analysis for clinical outcome of ICU admission and mechanical ventilation. Compared with the Delta variant, the Omicron variant was associated with a reduced risk for ICU admission (RR = 0.35, 95% CI: 0.29–0.42; RD% = 3.05, 95%CI: 2.59–3.51; Figure 2C; Table 2) and mechanical ventilation use (RR = 0.33, 95% CI: 0.25–0.44; RD% = 4.93, 95%CI: 4.04–5.83; Figure 2D; Table 2).

### 3.4. The reduction in hospitalized of patients with Omicron compared to Delta was more evident in older age groups

The hospitalization rate in the younger age group did not significantly differ between the two variants, but a strong effect was observed in the elderly group (Table 3; Supplementary Figure 1). The statistically significant upward trend in the absolute risk differences was observed in the three age subgroups (RD%: Younger: 0.69, 95%CI: 0.31–1.06; Medium: 2.42, 95%CI: 1.98–2.86; Elder: 10.61, 95%CI: 8.64–12.59; Supplementary Table S7). The reduction in relative risk of death with Omicron infections, as compared with Delta infections, was not age dependent (Table 3; Supplementary Figure 2). However, a significant increase in the absolute risk differences was observed in the elderly (RD%: Younger: 0.24, 95%CI: 0.00–0.49; Medium: 1.39, 95%CI: 1.23–1.56; Elder: 5.60, 95%CI: 4.65–6.55; Supplementary Table S7).

**Table 3:** Subgroup analysis by age (Omicron vs. Delta).

Age group	Hospitalized		Death	
	Studies	RR (95% CI)	Studies	RR (95% CI)
Younger	10	0.71 (0.47–1.06)	4	0.43 (0.21–0.86)
Medium	14	0.49 (0.40–0.60)	15	0.35 (0.29–0.41)
Elder	12	0.47 (0.42–0.53)	9	0.44 (0.36–0.54)
Overall	18	0.53 (0.46–0.61)	18	0.39 (0.34–0.44)

### 3.5. The risk of hospitalization of patients with Omicron compared to Delta decreased more sharply in booster vaccination group

**Figure 3:**
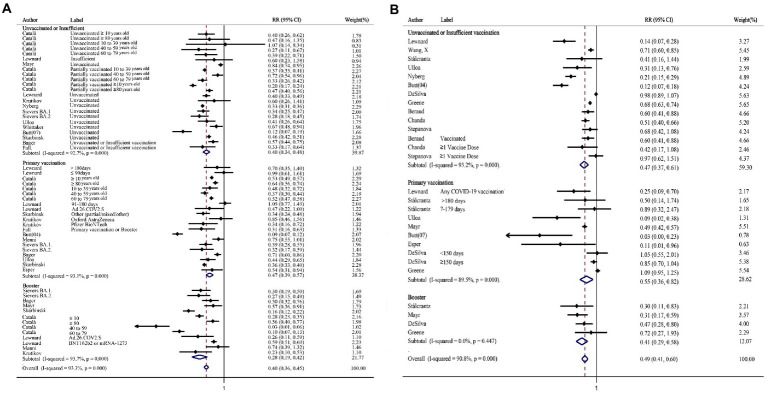
The forest plots of subgroups stratified by vaccination status (Omicron vs. Delta). **(A)** Risk ratio of hospitalization. **(B)** Risk ratio of death.

In the subgroup for hospitalization stratified by vaccination status, the relative risk ratio after Omicron infection was significantly reduced compared with that after the Delta infection, and the trend was declined significantly in the booster vaccination group compared with the other two groups (Figure 3A). Similarly, the absolute difference was also increased significantly after booster (RD%: Unvaccinated or Insufficient vaccinated: 4.36, 95%CI: 3.40–5.31; Primary vaccinated: 3.04, 95%CI: 2.22–3.85; Booster: 8.60, 95%CI: 5.95–11.24; Supplementary Table S7). The same trend of relative risk ratio reduction was seen in both mRNA vaccine group and adenovirus vaccine group (Supplementary Figure 3). The relative risk ratio of death decreased in all the subgroups stratified by vaccination status (Figure 3B). Concurrently, the absolute risk differences of death showed the same trend (RD%: Unvaccinated or Insufficient vaccinated: 1.90, 95%CI: 0.75–3.04; Primary vaccinated: 1.81, 95%CI: 0.81–2.80; Booster: 3.70, 95%CI: 0.34–7.06; Supplementary Table S7). Based on the included studies, the subgroup was performed in people who had received the mRNA vaccine, the relative risk ratio of death also decreased statistically (RR = 0.57, 95% CI: 0.46–0.70).

### 3.6. Analysis of subgroups stratified by patient source

The relative risk ratio declined more markedly in the general population (Supplementary Figure 4), and the absolute risk differences changed more sharply in the others (outpatient and inpatient, Supplementary Table S7).

### 3.7. Analysis of subgroups stratified by patient area

The relative risk ratio reductions for both analyses of hospitalization and death were greatest in studies based on Asia compared with other regions (Supplementary Table S8).

### 3.8. Sensitivity analysis and publication bias

In the sensitivity analysis of the four outcomes, the pooled RRs were similar before and after the removal of each study, indicating the stability of the current result (Supplementary Figure 5). Begg’s test showed no evident indication of publication bias, and the Funnel plots suggested no evidence of publication bias among the studies (Supplementary Figure 6).

## 4. Discussion

COVID-19 is an infectious disease that is pandemic in the world and brings great pressure to the public health systems in various countries (Baker et al., 2022). Given the continuous variation of new variants, clarifying the epidemiological characteristics of the current epidemic variants for further precise control and concentration of potential high-risk groups is particularly important. In the current study, we conducted a meta-analysis of epidemiological studies on Delta vs. Omicron variant in countries and regions worldwide, focusing on their absolute and relative risks in terms of hospitalization, mortality, ICU admission, and mechanical ventilation among infected people to provide high-level evidence for the formulation of more accurate epidemic prevention policies.

Almost all original studies showed that the risk of serious clinical outcomes caused by the Omicron variant decreased, as compared with the Delta variant, but the specific results reported by the different studies were inconsistent. Our meta-analysis data showed that the risk of serious clinical outcomes for the Omicron variant was down by half to two-thirds compared with the Delta variant. The decline in these risks could be attributed to the individual’s altered immune response caused by mutations in the virus itself. The main mutation site of the Omicron variant is in the spike protein of the virus, which causes significant changes in the variant transmissibility and disease severity (Bansal and Kumar, 2022). Compared with the pre-epidemic Delta variant, dozens of mutations make its epidemiological characteristics to have a lot of uncertainty, which needs the support of a wide range of global data (World Health Organization, 2021b). Several original studies have confirmed that the infectivity of the Omicron variant is significantly stronger than that of the Delta variant. Despite the fact that the effectiveness of the Omicron variant is significantly stronger than that of the Delta variant (Meo et al., 2021; World Health Organization, 2021b; Lewnard et al., 2022), after summarizing the data reported in different regions, we found that the relative risk of hospitalization and death caused by Omicron variant decreased significantly in Asia, Africa, Europe and the Americas. The relative risk ratio reductions for both analyses of hospitalization and death were greatest in studies based on Asia compared with other regions. This result needs to be confirmed with a larger sample size due to the small number of Asian studies included and two articles from the same author (Butt et al., 2022a,c; Intawong et al., 2023). However, regarding age, the main infected population of the Omicron variant has changed from middle-aged and elderly people to young people, as compared with the Delta variant (Meo et al., 2021; Shang et al., 2022). Therefore, although the absolute number of infections has increased significantly, the proportion of potential vulnerable populations that may have severe clinical outcomes has decreased. Moreover, the Omicron variants clinical symptoms are mainly mild, including headache, myalgia, fatigue, and cough (Malahe et al., 2023). Contrarily, the current basic research found that the Omicron variant lacks the functional region of the ACE2 receptor on the surface of human cells found in Delta variant due to genome mutation, which may be an important reason for the mild infection caused by the Omicron variant (Shah and Woo, 2021; Quarleri et al., 2022). The ability of the Omicron variant to cause cell fusion between infected cells is significantly lower than that of the Delta variant, and its replication ability is poor (Kandeel et al., 2022). Similarly, in patients with chronic diseases (with hypertension and diabetes), the risk of severe clinical outcomes caused by the Omicron variant is also significantly lower than that of the Delta variant. In addition to virus variation, reinfection leads to enhancement of individual immunity, and herd immunity is partly reached by a pandemic. Public health responses, including isolation and vaccination, and standardized domiciliary intervention guidance further reduce the patient’s hospitalization risk. The application of new drugs and improvement of clinical treatment also contributed to the reduction in severe clinical outcomes.

Although the hospitalization rate and relative risk of serious clinical events of the Omicron variant have decreased compared with the Delta variant, whether the degree of decline is consistent in different populations has important guiding significance for concise public health strategies related to COVID-19 in different populations. Additionally, in different populations, the decline in absolute risk is an important basis for health economic strategies. Age is the most important demographic factor. The younger the patients infected with the Omicron variant, the less obvious the decline in the hospitalization rate, as compared with the Delta variant. This may be related to the fact that COVID-19 vaccination in children has not been implemented on a large scale (Committee on Infectious Diseases, 2022). Contrarily, the decline in both the relative and absolute risks in hospitalization was most significant in the elderly group. In the epidemic of the Alpha and Delta variants, the infection rate and risk of severe clinical events of the elderly is relatively higher. Therefore, at the public health sector level, the government has vigorously promoted the primary and booster vaccinations in the older population (Arbel et al., 2021; Barda et al., 2021; Kiss et al., 2022). On an individual level, the elderly is more aware of their infection risk and more likely to comply with public health guidance and pay attention to personal health management than younger people (Hadjistavropoulos and Asmundson, 2022), contributing to a further reduction in hospitalization risk among elderly in the epidemic of the Omicron variant. In the elderly, the benefits from vaccination and higher health concern seem not to be effective in severe events. Therefore, the reduction of the relative risk of death in the elderly did not different from the other age groups. For risk of infection-related death, systemic inflammation induced by SARS-CoV-2 infection since chronic disease may play a greater role. This trend is consistent with the death risk of people from different sources. Compared with the general population, outpatient, emergency, and hospitalized patients have a significantly reduced risk for death after infected with Delta or Omicron variant. Although the hospitalization and severe clinical events rates of the Omicron variant are significantly lower than those of the Delta variant, for the elderly and vulnerable individuals with chronic diseases, attention should still be paid to the inflammatory chain reaction caused by various underlying diseases after developing a SARS-CoV-2 infection.

Vaccination is among the major public health strategies to cope with the COVID-19 pandemic worldwide. However, given the continuous mutation of the virus, whether to continue the vaccination of the existing vaccines or booster or develop new vaccines still needs to be fully explored. The protective efficacy of SARS-CoV-2 mRNA vaccine and live attenuated vaccine against the Omicron variant has been proven to be considerably reduced (Li, 2022). The current mRNA vaccine mainly targets the spike protein of SARS-CoV-2, while the main mutation site of the Omicron variant happens to be in the spike protein, supporting the decline in the protective effect of COVID-19 vaccine (Greaney et al., 2021). Concurrently, the variation of the Omicron variant can lead to the escape of antibodies induced by the patients themselves after an infection and the antibodies induced by the application of COVID-19 vaccine, which is more obvious than the Delta variant (Siddle et al., 2022). Therefore, previously infected or vaccinated individuals will still develop reinfections. A previous meta-analysis showed that, during the first 3 months of the Omicron wave, the reinfection rate reached 3.31%(Flacco et al., 2022). However, fortunately, the reinfection rate is still significantly lower in the vaccinated individuals than in those without vaccination (0.32% vs. 0.74%). Although vaccination had lost its effectiveness against the Omicron infection, it still provided significant additional protection against COVID-19-related hospitalization and death. Especially, booster vaccination may produce many neutralizing antibodies in the body, considerably enhancing the vaccines protective effect against the Omicron variant (Kiss et al., 2022). In a recent study on the Omicron variant, the relative risk of death of the population vaccinated with one booster dose is decline 82% than that of the non-vaccinated population, and it dropped by 99% after receiving two booster doses (Kiss et al., 2022). Therefore, more population-based studies investigating whether it is necessary to improve the booster vaccination strategy are warranted to provide more powerful evidence on the efficacy of booster vaccination. From our meta-analysis, regardless of whether the patients are vaccinated or not, the hospitalization and mortality rates of the Omicron variant, compared with the Delta variant, is decreased. Moreover, there was significant difference in the degree of reduction of the relative risk between booster and other vaccination groups, indicating that, in the event of hospitalization and death, intensive vaccination did have an interaction with different variants. The change degree of the absolute risk is the highest in the booster group. Therefore, our research results can be used as evidence to confirm even if the virulence of Omicron variant has decreased significantly compared with Delta variant, booster vaccination of the current vaccine or further vaccination against Omicron variant is necessary to reduce the medical burden of the public health system and improve the possible adverse outcomes after infection.

Our study has several limitations. First, most of the original studies included only reported the relative risk after controlling covariates. To further explore the absolute reduction of medical burden caused by the Omicron variant compared with the Delta variant, we used the original four-grid table to calculate the rate difference under a single factor, which may have a certain bias. Second, we did not find any relevant research conducted in East Asia. Therefore, whether our results would vary in this racial group needs further investigation. Third, as different countries and regions may have different epidemic prevention policies and medical intervention standards for COVID-19, the indicators of hospitalization may be biased. Finally, there has certain bias in treating OR and HR as similar RR. The directionality of this bias has been shown in the subgroup analysis. Thus, we should reasonably select effect indicators when conducting prospective studies related to COVID-19.

In conclusion, although the ability of the Omicron variant to cause hospitalization and adverse events has decreased significantly, as compared with the Delta variant, vulnerable populations need to still be vigilant. Concurrently, vaccination is still an effective means of protection. Continuous and systematic tracking of virus mutations is necessary. How to balance the consumption of public health resources and economic development is still a long-term question.

## Data availability statement

The original contributions presented in the study are included in the article/Supplementary material, further inquiries can be directed to the corresponding author.

## Author contributions

JJ, YW, and YP contributed to the study conception and design. YW and YP performed the literature search. YW, YP, JY, HL, LZ, and MX performed data extraction and quality evaluation. YZ, KS, and ZJ analyzed the data. DC, JY, and HL prepared figures and tables. YW, YP, KS, and YZ contributed to the article writing. JJ, YW, and YP made the final decision. All authors contributed to the article and approved the submitted version.

## Funding

This study was supported by the Youth Development Fund from First Hospital of Jilin University (JDYY11202124 and JDYY11202128), the Education Department of Jilin Province (JJKH20211153KJ).

## Conflict of interest

The authors declare that the research was conducted in the absence of any commercial or financial relationships that could be construed as a potential conflict of interest.

## Publisher’s note

All claims expressed in this article are solely those of the authors and do not necessarily represent those of their affiliated organizations, or those of the publisher, the editors and the reviewers. Any product that may be evaluated in this article, or claim that may be made by its manufacturer, is not guaranteed or endorsed by the publisher.
