# Supplementary material for: Elder and booster vaccination associates with decreased risk of serious clinical outcomes in comparison of Omicron and Delta variant: A meta-analysis of SARS-CoV-2 infection
Source: Front Microbiol. 2023 Apr 14;14:1051104. doi: 10.3389/fmicb.2023.1051104 (PMC10140352; doi:10.3389/fmicb.2023.1051104)
Supplement: Supplementary file 1 [file Data_Sheet_1.doc]

**Supplementary Appendix**

**Supplementary Table1.** Newcastle–Ottawa Scale(NOS) Quality Assessment Table.

**Supplementary Table2.** Eligible studies of clinical outcome of hospitalization**.**

**Supplementary Table3.** Eligible studies of clinical outcome of death.

**Supplementary Table4.** Eligible studies of clinical outcome of ICU admission.

**Supplementary Table5.** Eligible studies of clinical outcome of mechanical ventilation.

**Supplementary Table6.** Pooled RR of the subgroup analysis for the clinical outcome of hospitalization, death, stratified by effect index.

**Supplementary Table7.** Pooled RD of the subgroup analysis for the clinical outcome of hospitalization and death.

**Supplementary Table8.** Pooled RR of the subgroup analysis for the clinical outcome of hospitalization and death, stratified by patient areas

**Supplementary Figure1.** The forest plots of subgroups for risk of hospitalization stratified by age (Omicron *vs.*Delta).

**Supplementary Figure2.** The forest plots of subgroups for risk of death stratified by age (Omicron *vs.*Delta).

**Supplementary Figure3.** The forest plots of subgroups for risk of death stratified by patient source (Omicron vs.Delta).

**Supplementary Figure4.** The forest plots of subgroups for risk of death stratified by patient source (Omicron vs.Delta).

**Supplementary Figure5.** Sensitivity analysis for risk of (A) hospitalization. (B) death. (C) ICU admission.(D) mechanical ventilation.

**Supplementary Figure6.** Funnel plot for risk of (A) hospitalization. (B) death. (C) ICU admission.(D) mechanical ventilation.

**Supplementary Table1. Newcastle-Ottawa Scale(NOS) Quality Assessment Table**

| **No** | **Author** | **Selection** | **Comparability** | **Outcome** | **Score** |
| --- | --- | --- | --- | --- | --- |
|
| 1 | Wang L | 4 | 2 | 3 | 9 |
|
| 2 | Kahn | 4 | 2 | 2 | 8 |
|
| 3 | Whittaker | 4 | 2 | 2 | 8 |
|
| 4 | Pascall | 4 | 2 | 2 | 8 |
|
| 5 | Wang X | 4 | 2 | 1 | 7 |
|
| 6 | Ulloa | 4 | 2 | 3 | 9 |
|
| 7 | Butt (2022.04) | 4 | 1 | 3 | 8 |
|
| 8 | Wrenn | 4 | 2 | 2 | 8 |
|
| 9 | Menni | 4 | 2 | 2 | 8 |
|
| 10 | Nyberg | 4 | 2 | 3 | 9 |
|
| 11 | Shi | 4 | 2 | 1 | 7 |
|
| 12 | Krutikov | 4 | 2 | 2 | 8 |
|
| 13 | Sacco | 3 | 2 | 2 | 7 |
|
| 14 | Vieillard  Baron | 4 | 2 | 1 | 7 |
|
| 15 | Fall | 4 | 2 | 2 | 8 |
|
| 16 | Butt (2022.05) | 4 | 2 | 3 | 9 |
| 17 | Lewnard | 4 | 2 | 3 | 9 |
|
| 18 | Bouzid | 4 | 2 | 2 | 8 |
|
| 19 | Davies | 4 | 2 | 2 | 8 |
|
| 20 | Sievers | 4 | 2 | 2 | 8 |
|
|
| 21 | Auvigne | 4 | 2 | 3 | 9 |
|
| 22 | Stålcrantz | 3 | 2 | 2 | 7 |
|
| 23 | Van | 3 | 1 | 3 | 7 |
|
| 24 | Skarbinski | 4 | 2 | 3 | 9 |
|
| 25 | Mayr | 4 | 2 | 3 | 9 |
|
|
| 26 | Jassat | 3 | 2 | 3 | 8 |
|
| 27 | Bager | 4 | 2 | 3 | 9 |
|
| 28 | Goga | 4 | 2 | 3 | 9 |
|
| 29 | Butt(2022.07) | 4 | 1 | 3 | 7 |
|
| 30 | Greene | 4 | 2 | 3 | 9 |
| 31 | Wolter | 4 | 2 | 3 | 9 |
| 32 | Bonsignore | 4 | 1 | 3 | 8 |
| 33 | Stepanova | 4 | 2 | 3 | 9 |
| 34 | Català | 4 | 2 | 3 | 9 |
| 35 | Esper | 4 | 2 | 2 | 8 |
| 36 | Strasser | 4 | 2 | 3 | 9 |
| 37 | Intawong | 4 | 2 | 2 | 8 |
| 38 | DeSilva | 4 | 2 | 2 | 8 |
| 39 | Nevejan | 4 | 1 | 3 | 8 |
| 40 | Beraud | 4 | 2 | 3 | 8 |
| 41 | Chanda | 4 | 1 | 2 | 7 |
| 42 | Adjei | 4 | 1 | 3 | 8 |
| 43 | Trobajo | 4 | 1 | 3 | 8 |

**Supplementary Table2. E**ligible studies of clinical outcome of hospitalization

| **No** | **Author** | **Region** | **Population** | **Ages** | **Sample size** | **Effect index** | **Effect index**  **value** |
| --- | --- | --- | --- | --- | --- | --- | --- |
| 1 | Wang L | USA | General population | All | D:147,107 | RR | 0.58 (0.55–0.60) |
|  |  |  |  |  | O:147,107 |  |  |
| 2 | Whittaker | Norway | General children | <18 | D:42,362 | RR | 0.67 (0.48–0.94) |
|  |  |  |  |  | O:82,907 |  |  |
| 3 | Pascall | UK | General population | ≥18 | D:1,164 | RR | 0.47 (0.26-0.84)* |
|  |  |  |  |  | O:2,694 |  |  |
| 4 | Ulloa | Canada | General population | All | D:9,087 | HR | 0.41 (0.30-0.55) |
|  |  |  |  |  | O:9,087 |  |  |
| 5 | Butt(2022.04) | Qatar | General population | <18 | D:985 | OR | 0.12 (0.07-0.19) |
|  |  |  |  |  | O:985 |  |  |
| 6 | Wrenn | USA | General population | All | D:489 | OR | 0.40 (0.21–0.78) |
|  |  |  |  |  | O:263 |  |  |
| 7 | Menni | UK | General population,  at least 2 doses of vaccine | ≥16 | D:4,990 | OR | 0·75 (0·57–0·98) |
|  |  |  |  |  | O:4,990 |  |  |
| 8 | Nyberg | UK | General population | All | D:448,843 | HR | 0.41 (0.39–0.43) |
|  |  |  |  |  | O:1,067,859 |  |  |
| 9 | Krutikov | UK | Rresidents of Long-Term Care Facilities | ≥65 | D:400 | HR | 0.64 (0.41–1.00) |
|  |  |  |  |  | O:1,864 |  |  |
| 10 | Sacco | Italy | Individuals with at most one episode of reinfection | All | D:6,030 | IRR | 0.37 (0.30-0.46) |
|  |  |  |  |  | O:163,468 |  |  |
| 11 | Fall | USA | Inpatients and Outpatients | All | D:908 | OR | 0.34 (0.21-0.53) |
|  |  |  |  |  | O:1,119 |  |  |
| 12 | Butt(2022.05) | USA | General population | ＞20 | D:2619 | RR | 0.52(0.46-0.59) |
|  |  |  |  |  | O: 18906 |  |  |
| 13 | Lewnard | USA | General population | All | D:23,305 | HR | 0.59 (0.51-0.69) |
|  |  |  |  |  | O:222,688 |  |  |
| 14 | Bouzid | France | Emergency patient | ≥16 | D:818 | AR | 0.78 (0.64-0.94)* |
|  |  |  |  |  | O:898 |  |  |
| 15 | Davies | South Africa | General population | ≥20 | D:4,355 | RR | 0.72 (0.63-0.82) |
|  |  |  |  |  | O:5,104 |  |  |
| 16 | Sievers | Germany | General population | All | D:24,530 | OR | 0.35 (0.29–0.43) |
|  |  |  |  |  | BA.1:163,468 |  |  |
|  |  |  |  |  | BA.2:6,860 |  | 0.30 (0.22–0.40) |
| 17 | Skarbinski | USA | General population | All | D:69,977 | HR | 0.55 (0.51-0.59) |
|  |  |  |  |  | O:48,101 |  |  |
| 18 | Mayr | USA | Veterans | ≥18 | D:22,841 | RR | 0.89 (0.83-0.96) |
|  |  |  |  |  | O:22,841 |  |  |
| 19 | Jassat | South Africa | General population | All | D:1,306,260 | RR | 0.82 (0.81-0.83)* |
|  |  |  |  |  | O:629,617 |  |  |
| 20 | Bager | Denmark | General population | All | D:150,311 | RR | 0·64 (0.56–0.75) |
|  |  |  |  |  | O:38,669 |  |  |
| 21 | Goga | South Africa | General population | ≥18 | D:15,195 | OR | 0.60 (0.54-0.66) |
|  |  |  |  |  | O:26,393 |  |  |
| 22 | Butt(2022.07) | Qatar | General population | ≥18 | D:3,926 | OR | 0.07(0.05-0.09) |
|  |  |  |  |  | O:3,926 |  |  |
| 23 | Greene | USA | General population | All age | D:158799 | RR | 0.72 (0.63, 0.82) |
|  |  |  |  |  | O:488053 |  |  |
| 24 | Wolter | South Africa | General population | All age | D:1273 | RR | 0.29(0.25-0.39) |
|  |  |  |  |  | BA.1: 75,763 |  |  |
|  |  |  |  |  |  |  | 0.24(0.20-0.29) |
|  |  |  |  |  | BA.2: 20,068 |  |  |
|  |  |  |  |  |  |  | 0.36(0.27-0.47) |
|  |  |  |  |  | BA.4/BA.5: 1806 |  |  |
| 25 | Català | Spain | General population | ＞10 | D:997,748 | RR | 0.46 ( 0.43- 0.49) |
|  |  |  |  |  | O:11,121,316 |  |  |
| 26 | Esper | America | General population | All ages | D: 808 | RR | 0.46(0.32-0.67) |
|  |  |  |  |  | O: 696 |  |  |
| 27 | Strasser | England | General population | All age | D:1223 | RR | 0.53（0.48-0.58） |
|  |  |  |  |  | O: 911 |  |  |
| 28 | Trobajo | Navarra, Spain. | General population | All age | D: 487 | OR | 0.28 (0.16-0.47) |
|  |  |  |  |  | O:1867 |  |  |

*: Caculated from oringnal N

**Supplementary Table3. Eligible studies of clinical outcome of death**

| **No** | **Author** | **Region** | **Population** | **Ages** | **Samplesize** | **Effect index** | **Effect index**  **value** |
| --- | --- | --- | --- | --- | --- | --- | --- |
| 1 | Wang X | USA | General population | All | D:27,001 | OR | 0.56 (0.49-0.63) |
|  |  |  |  |  | O:45,223 |  |  |
| 2 | Ulloa | Canada | General population | All | D:9,087 | HR | 0.12 (0.04-0.37) |
|  |  |  |  |  | O:9,087 |  |  |
| 3 | Butt(2022.04) | Qatar | General population | <18 | D:985 | OR | 0.12 (0.07-0.18) |
|  |  |  |  |  | O:985 |  |  |
| 4 | Wrenn | USA | General population | All | D:489 | / | Incalculable |
|  |  |  |  |  | O:263 |  |  |
| 5 | Nyberg | UK | General population | All | D:448,843 | HR | 0.31 (0.26–0.37) |
|  |  |  |  |  | O:1,067,859 |  |  |
| 6 | Shi | USA | Hospitalized children | 5-11 | D:482 | / | Incalculable |
|  |  |  |  |  | O:397 |  |  |
| 7 | Krutikov | UK | Rresidents of Long-Term Care Facilities | ≥65 | D:400 | HR | 0.68 (0.44–1.04) |
|  |  |  |  |  | O:1,864 |  |  |
| 8 | Sacco | Italy | Individuals with at most one episode of reinfection | All | D:6,030 | IRR | 0.37 (0.30-0.46) |
|  |  |  |  |  | O:163,468 |  |  |
| 9 | Vieillard | France | Hospitalized patients | All | D:400 | RR | 0.72 (0.45-1.14)* |
|  |  |  |  |  | O:229 |  |  |
| 10 | Fall | USA | Inpatients and Outpatients | All | D:908 | OR | 0.39 (0.17-0.89) |
|  |  |  |  |  | O:1,119 |  |  |
| 11 | Butt(2022.05) | USA | General population | ＞20 | D:2619 | RR | 0.51(0.29-0.87)* |
|  |  |  |  |  | O: 18906 |  |  |
| 12 | Lewnard | USA | General population | All | D:23,305 | HR | 0.21 (0.10-0.44) |
|  |  |  |  |  | O:222,688 |  |  |
| 13 | Bouzid | France | Emergency patient | ≥16 | D:818 | AR | 0.42 (0.28-0.64)* |
|  |  |  |  |  | O:898 |  |  |
| 14 | Davies | South Africa | General population | ≥20 | D:4,355 | RR | 0.41 (0.29-0.59) |
|  |  |  |  |  | O:5,104 |  |  |
| 15 | Sievers | Germany | General population | All | D:24,530 | OR | 0.38 (0.25–0.58) |
|  |  |  |  |  | BA.1:163,468 |  |  |
|  |  |  |  |  | BA.2:6,860 |  | 0.16 (0.08–0.30) |
| 16 | Auvigne | France | General population | ≥18 | D:92,182 | HR | 0.12 (0.08-0.18) |
|  |  |  |  |  | O:92,182 |  |  |
| 17 | Stålcrantz | Norway | Hospitalized patients | All | D:666 | HR | 0.44 (0.24–0.79) |
|  |  |  |  |  | O:409 |  |  |
| 18 | Van | Belgium | Hospitalized patients | ≥18 | D:509 | RR | 0.78 (0.28; 1.29) |
|  |  |  |  |  | O:445 |  |  |
| 19 | Skarbinski | USA | General population | All | D:69,977 | HR | 0.54 (0.42-0.70) |
|  |  |  |  |  | O:48,101 |  |  |
| 20 | Mayr | USA | Veterans | ≥18 | D:22,841 | RR | 0.42 (0.39-0.46) |
|  |  |  |  |  | O:22,841 |  |  |
| 21 | Jassat | South Africa | General population | All | D:1,306,260 | OR | 0.29 (0.28-0.30) |
|  |  |  |  |  | O:629,617 |  |  |
| 22 | Butt(2022.07) | Qatar | General population | ≥18 | D:3,926 | OR | 0.02 (0.00-0.14) |
|  |  |  |  |  | O:3,926 |  |  |
| 23 | Greene | USA | General population | All | D:158799 | RR | 0.81 (0.58, 1.13) |
|  |  |  |  |  | O:488053 |  |  |
| 24 | Wolter | South Africa | General population | All | D:1273 | RR | 0.58（0.43-0.80） |
|  |  |  |  |  | BA.1:75,763 |  |  |
|  |  |  |  |  |  |  | 0.45（0.32-0.65） |
|  |  |  |  |  | BA.2:20,068 |  |  |
|  |  |  |  |  |  |  | 0.48（0.27-0.85） |
|  |  |  |  |  | BA.4/BA.5: 1806 |  |  |
| 25 | Bonsignore | Germany | Hospitalized patients | ≥18 | D:12,370 | OR | 0.43(0.40-0.49)* |
|  |  |  |  |  | O:21,222 |  |  |
| 26 | Stepanova. | USA | Hospitalized patients | ≥18 | D:860 | RR | 0.82(0.61-1.10) |
|  |  |  |  |  | O:1556 |  |  |
| 27 | Esper | America | General population | All | D:808 | RR | 0.44(0.12-1.65) |
|  |  |  |  |  | O:696 |  |  |
| 28 | Strasser | England | Hospitalized patients | All | D:20770 | RR | 0.36（0.27-0.48） |
|  |  |  |  |  | O:28940 |  |  |
| 29 | Intawong | Thailand | General population | ≥18 | D:17047 | HR | 0.09(0.07-0.11) |
|  |  |  |  |  | O:188043 |  |  |
| 30 | DeSilva | USA | Hospitalized patients | ≥18 | D:16078 | RR | 0.59(0.54-0.64) |
|  |  |  |  |  | O:11071 |  |  |
| 31 | Nevejan | Belgium | Hospitalized patients | ≥18 | D:187 | OR | 0.24(0.14–0.40) |
|  |  |  |  |  | O:1036 |  |  |
| 32 | Beraud | Bulgaria, Croatia, France, Turkey | Hospitalized patients | All | D:955 | OR | 0.53 (0.37; 0.76) |
|  |  |  |  |  | O:1215 |  |  |
| 33 | Chanda | Zambia | Hospitalized patients | All | D:752 | OR | 0.42(0.32-0.53) |
|  |  |  |  |  | O: 901 |  |  |
| 34 | Adjei | USA | Hospitalized patients | All | D:163,094 | RR | 0.69(0.68 to 0.70) |
|  |  |  |  |  | Early O: 104,395 |  |  |
|  |  |  |  |  |  |  | 0.24(0.22 to 0.25) |
|  |  |  |  |  | Later O:20,655 |  |  |
| 35 | Trobajo | Navarra, Spain. | General population | All | D: 487 | OR | 0.23 (0.12-0.46) |
|  |  |  |  |  | O: 1867 |  |  |

*: Caculated from oringnal N

**Supplementary Table4 Eligible studies of clinical outcome of** ICU admission

| **No** | **Author** | **Region** | **Population** | **Ages** | **Samplesize** | **Effect index** | **Effect index**  **value** |
| --- | --- | --- | --- | --- | --- | --- | --- |
| 1 | Wang L | USA | General population | All | D:147,107 | RR | 0.47 (0.43–0.51) |
|  |  |  |  |  | O:147,107 |  |  |
| 2 | Kahn | Sweden | General population | All | D:13,711 | RR | 0.29 (0.24-0.37)* |
|  |  |  |  |  | O:29,539 |  |  |
| 3 | Ulloa | Canada | General population | All | D:9,087 | HR | 0.19 (0.09-0.39) |
|  |  |  |  |  | O:9,087 |  |  |
| 4 | Butt(2022.04) | Qatar | General population | <18 | D:985 | OR | 0.12 (0.07-0.18) |
|  |  |  |  |  | O:985 |  |  |
| 5 | Shi | USA | Hospitalized children | 5-11 | D:482 | RR | 0.47 (0.32-0.68) |
|  |  |  |  |  | O:397 |  |  |
| 6 | Fall | USA | Inpatients and Outpatients | All | D:908 | OR | 0.22 (0.05-0.91) |
|  |  |  |  |  | O:1,119 |  |  |
| 7 | Butt(2022.05) | USA | General population | ＞20 | D:2619 | RR | 0.51(0.29-0.87)* |
|  |  |  |  |  | O: 18906 |  |  |
| 8 | Lewnard | USA | General population | All | D:23,305 | HR | 0.48 (0.29-0.81) |
|  |  |  |  |  | O:222,688 |  |  |
| 9 | Bouzid | France | Emergency patient | ≥16 | D:818 | RR | 0.24 (0.17-0.35)* |
|  |  |  |  |  | O:898 |  |  |
| 10 | Davies | South Africa | General population | ≥20 | D:4,355 | RR | 0.43 (0.33-0.55) |
|  |  |  |  |  | O:5,104 |  |  |
| 11 | Sievers | Germany | General population | All | D:24,530 | OR | 0.20 (0.12–0.32) |
|  |  |  |  |  | BA.1:163,468 |  |  |
|  |  |  |  |  | BA.2:6,860 |  | 0.17 (0.07–0.39) |
| 12 | Auvigne | France | General population | ≥18 | D:92,182 | HR | 0.12 (0.08-0.18) |
|  |  |  |  |  | O:92,182 |  |  |
| 13 | Stålcrantz | Norway | Hospitalized patients | All | D:666 | HR | 0.52 (0.34–0.80) |
|  |  |  |  |  | O:409 |  |  |
| 14 | Van | Belgium | Hospitalized patients | ≥18 | D:509 | RR | 0.78 (0.28-1.29) |
|  |  |  |  |  | O:445 |  |  |
| 15 | Mayr | USA | Veterans | ≥18 | D:22,841 | RR | 0.42 (0.39-0.46) |
|  |  |  |  |  | O:22,841 |  |  |
| 16 | Jassat | South Africa | General population | All | D:1,306,260 | OR | 0.29 (0.28-0.30) |
|  |  |  |  |  | O:629,617 |  |  |
| 17 | Goga | South Africa | General population | ≥18 | D:15,195 | OR | 0.30 (0.19-0.47) |
|  |  |  |  |  | O:26,393 |  |  |
| 18 | Butt(2022.07) | Qatar | General population | ≥18 | D:3,926 | OR | 0.019 (0.003-0.143) |
|  |  |  |  |  | O:3,926 |  |  |
| 19 | Wolter | South Africa | General population | All | D:1273 | RR | 0.58（0.43-0.80） |
|  |  |  |  |  | BA.1: 75,763 |  |  |
|  |  |  |  |  |  |  | 0.45（0.32-0.65） |
|  |  |  |  |  | BA.2: 20,068 |  |  |
|  |  |  |  |  |  |  | 0.48（0.27-0.85） |
|  |  |  |  |  | BA.4/BA.5: 1806 |  |  |
| 20 | Bonsignore | Germany | Hospitalized patients | ≥18 | D: 12,370 | OR | 0.56(0.52-0.59)* |
|  |  |  |  |  | O: 21,222 |  |  |
| 21 | Stepanova. | USA | Hospitalized patients | ≥18 | D: 860 | RR | 0.71（0.57-0.88） |
|  |  |  |  |  | O: 1556 |  |  |
| 22 | Català | Spain | General population | ＞10 | D：997,748 | RR | 0.25(0.21- 0.28) |
|  |  |  |  |  | O:11,121,316 |  |  |
| 23 | Esper | America | General population | All | D: 808 | RR | 0.28(0.12-0.64) |
|  |  |  |  |  | O: 696 |  |  |
| 24 | Strasser | England | Hospitalized patients | All | D：20770 | RR | 0.36（0.28-0.46） |
|  |  |  |  |  | O：28940 |  |  |
| 25 | DeSilva | USA | Hospitalized patients | ≥18 | D: 16078 | RR | 0.59(0.55-0.63) |
|  |  |  |  |  | O: 11071 |  |  |
| 26 | Nevejan | Belgium | Hospitalized patients | ≥18 | D: 187 | OR | 0.26(0.17–0.40) |
|  |  |  |  |  | O: 1036 |  |  |
| 27 | Beraud | Bulgaria, Croatia, France, Turkey | Hospitalized patients | All | D：955 | OR | 0.19 (0.12; 0.28) |
|  |  |  |  |  | O:1215 |  |  |
| 28 | Adjei | USA | Hospitalized patients | All | D:163,094 | RR | 0.85(0.84-0.87) |
|  |  |  |  |  | Early O: 104,395 |  |  |
|  |  |  |  |  |  |  | 0.53(0.51-0.55) |
|  |  |  |  |  | Later O:20,655 |  |  |
| 29 | Trobajo | Navarra, Spain. | General population | All | D: 487 | OR | 0.23 (0.12-0.46) |
|  |  |  |  |  | O: 1867 |  |  |

*: Caculated from oringnal N

**Supplementary Table5. Eligible studies of clinical outcome of mechanical ventilation**

| **No** | **Author** | **Region** | **Population** | **Ages** | **Samplesize** | **Effect index** | **Effect index**  **value** |
| --- | --- | --- | --- | --- | --- | --- | --- |
| 1 | Wang L | USA | General population | All | D:147,107 | RR | 0.25 (0.20–0.31) |
|  |  |  |  |  | O:147,107 |  |  |
| 2 | Butt(2022.04) | Qatar | General population | <18 | D:985 | OR | 0.12 (0.07-0.18) |
|  |  |  |  |  | O:985 |  |  |
| 3 | Wrenn | USA | General population | All | D:489 | OR | 0.39 (0.17-0.87) |
|  |  |  |  |  | O:263 |  |  |
| 4 | Shi | USA | Hospitalized children | 5-11 | D:482 | RR | 0.42 (0.20-0.87)* |
|  |  |  |  |  | O:397 |  |  |
| 5 | Vieillard | France | Hospitalized patients | All | D:400 | RR | 0.80 (0.58-1.12) |
|  |  |  |  |  | O:229 |  |  |
| 6 | Lewnard | USA | General population | All | D:23,305 | HR | 0.36 (0.18-0.72) |
|  |  |  |  |  | O:222,688 |  |  |
| 7 | Bouzid | France | Emergency patient | ≥16 | D:818 | RR | 0.28 (0.16-0.49)* |
|  |  |  |  |  | O:898 |  |  |
| 8 | Davies | South Africa | General population | ≥20 | D:4,355 | RR | 0.43 (0.33-0.55) |
|  |  |  |  |  | O:5,104 |  |  |
| 9 | Van | Belgium | Hospitalized patients | ≥18 | D:509 | RR | 0.22 (0.10-0.49) |
|  |  |  |  |  | O:445 |  |  |
| 10 | Skarbinski | USA | General population | All | D:69,977 | HR | 0.43 (0.33-0.56) |
|  |  |  |  |  | O:48,101 |  |  |
| 11 | Mayr | USA | Veterans | ≥18 | D:22,841 | RR | 0.40 (0.33-0.49) |
|  |  |  |  |  | O:22,841 |  |  |
| 12 | Jassat | South Africa | General population | All | D:1,306,260 | OR | 0.29 (0.28-0.30) |
|  |  |  |  |  | O:629,617 |  |  |
| 13 | Goga | South Africa | General population | ≥18 | D:15,195 | OR | 0.12 (0.07-0.22) |
|  |  |  |  |  | O:26,393 |  |  |
| 14 | Butt(2022.07) | Qatar | General population | ≥18 | D:3,926 | OR | 0.02(0.00-0.14) |
|  |  |  |  |  | O:3,926 |  |  |
| 15 | Wolter | South Africa | General population | All | D:1273 | RR | 0.58（0.43-0.80） |
|  |  |  |  |  | BA.1: 75,763 |  |  |
|  |  |  |  |  |  |  | 0.45（0.32-0.65） |
|  |  |  |  |  | BA.2: 20,068 |  |  |
|  |  |  |  |  | BA.4/BA.5: 1806 |  |  |
|  |  |  |  |  |  |  | 0.48（0.27-0.85） |
| 16 | Bonsignore | Germany | Hospitalized patients | ≥18 | D: 12,370 | OR | 0.27(0.25-0.29)* |
|  |  |  |  |  | O: 21,222 |  |  |
| 17 | Stepanova. | USA | Hospitalized patients | ≥18 | D: 860 | RR | 0.56（0.43-0.74） |
|  |  |  |  |  | O: 1556 |  |  |
| 18 | Esper | America | General population | All | D: 808 | RR | 0.21(0.05-0.96) |
|  |  |  |  |  | O: 696 |  |  |
| 19 | Strasser | England | Hospitalized patients | All | D：20770 | RR | 0.46（0.36-0.59） |
|  |  |  |  |  | O：28940 |  |  |
| 20 | Intawong | Thailand | General population | ≥18 | D:17047 | HR | 0.08(0.06-0.10)* |
|  |  |  |  |  | O:188043 |  |  |
| 21 | DeSilva | USA | Hospitalized patients | ≥18 | D: 16078 | RR | 0.83(0.76-0.90) |
|  |  |  |  |  | O: 11071 |  |  |
| 22 | Beraud | Bulgaria, Croatia, France, Turkey | Hospitalized patients | All | D：955 | OR | 0.34(0.23-0.51)* |
|  |  |  |  |  | O:1215 |  |  |
| 23 | Adjei | USA | Hospitalized patients | All | D:163,094 | RR | 0.77(0.76-0.79) |
|  |  |  |  |  | Early O: 104,395 |  |  |
|  |  |  |  |  |  |  | 0.24(0.23-0.26) |
|  |  |  |  |  | Later O:20,655 |  |  |

*: Caculated from oringnal N

**Supplementary Table6. Pooled RR of the subgroup analysis for the clinical outcome of hospitalization, death, stratified by effect index**

| Subgroup | Studies (N) | Pooled RR (95% CI） |
| --- | --- | --- |
|
| Hospitalized |  |  |
| RR | 15 | 0.53(0.46-0.62) |
| HR | 5 | 0.50(0.41-0.61) |
| OR | 8 | 0.26(0.14-0.48) |
| Death |  |  |
| RR | 15 | 0.49(0.37-0.64) |
| HR | 8 | 0.25(0.14-0.45) |
| OR | 10 | 0.32(0.25-0.41) |

**Supplementary Table7. Pooled RD of the subgroup analysis for the clinical outcome of hospitalization and death**

|  | Hospitalized | | Death | |
| --- | --- | --- | --- | --- |
| Subgroup | Studies | RD%(95%CI) | Studies | RD%(95%CI) |
| Age |  |  |  |  |
| Younger | 9 | 0.69(0.31-1.06) | 7 | 0.24(0.00-0.49) |
| Medium | 12 | 2.42(1.98-2.86) | 10 | 1.39(1.23-1.56) |
| Elder | 10 | 10.61(8.64-12.59) | 7 | 5.60(4.65-6.55) |
| Overall | 16 | 3.62(3.23-4.02) | 14 | 2.08(1.91-2.25) |
| Vaccination |  |  |  |  |
| Unvaccinated or Insufficient vaccinated | 7 | 4.36(3.40-5.31) | 7 | 1.90(0.75-3.04) |
| Primary vaccinated | 7 | 3.04(2.22-3.85) | 9 | 1.81(0.81-2.80) |
| Booster | 6 | 8.60(5.95-11.24) | 5 | 3.70(0.34-7.06) |
| Overall | 9 | 4.35(3.83-4.87) | 10 | 1.13(0.76-1.50) |
| Patients source |  |  |  |  |
| General population | - | - | 16 | 2.13(1.72-2.54) |
| Others | - | - | 19 | 5.93(3.33-8.53) |
| Overall | - | - | 35 | 3.10(2.67-3.53) |

**Supplementary Table8.** Pooled RR of the subgroup analysis for the clinical outcome of hospitalization and death, stratified by patient areas

|  | Studies | RR(95%CI) |
| --- | --- | --- |
| Hospitalized |  |  |
| America | 9 | 0.58(0.50-0.68) |
| Europe | 12 | 0.49(0.43-0.57) |
| Africa | 5 | 0.44(0.32-0.62) |
| Asia | 2 | 0.09(0.05-0.15) |
| Overall | 28 | 0.45(0.39-0.0.52) |
| Death |  |  |
| America | 11 | 0.47(0.35-0.65) |
| Europe | 14 | 0.37(0.30-0.45) |
| Africa | 5 | 0.42(0.32-0.55) |
| Asia | 3 | 0.09(0.06-0.14) |
| Over all | 33 | 0.37(0.30-0.45) |


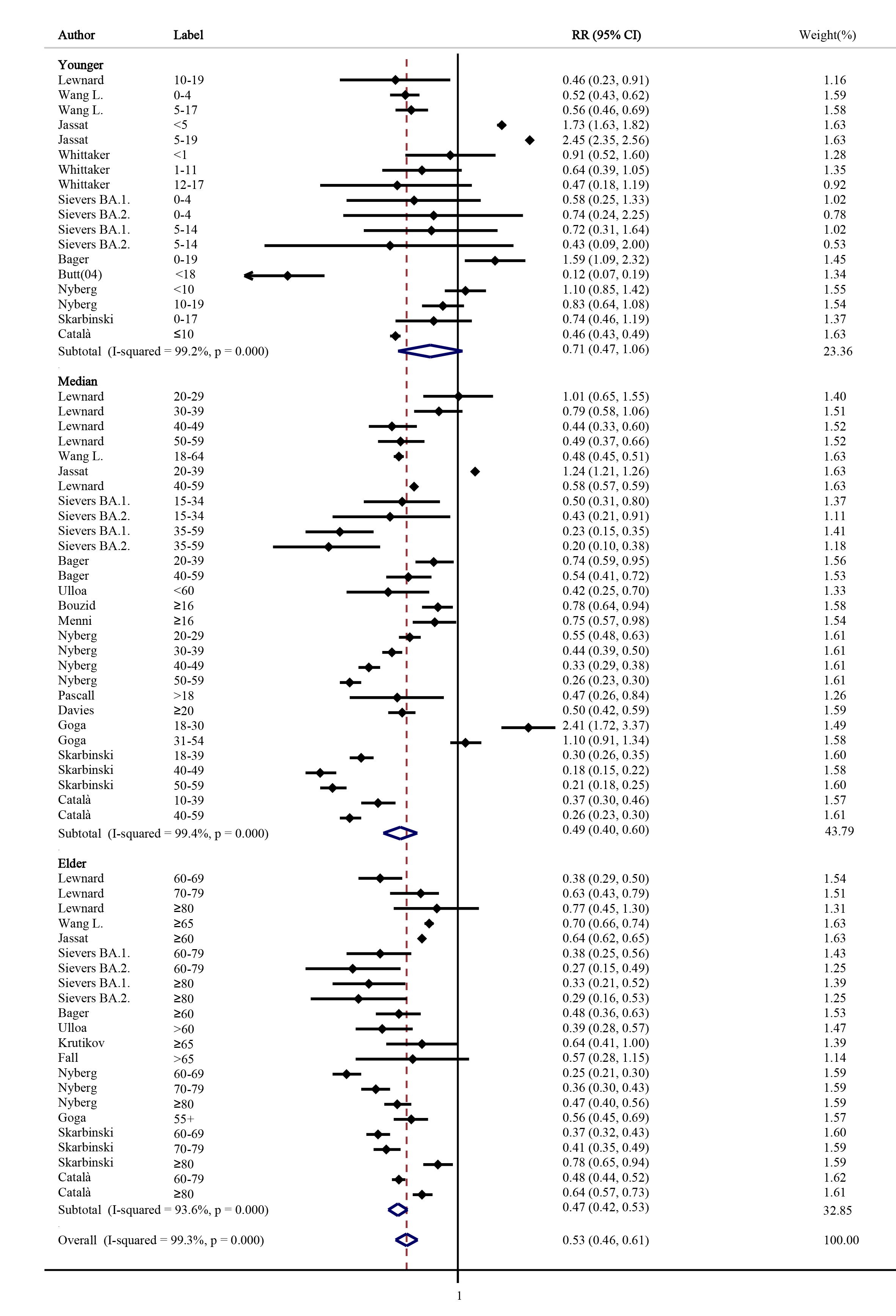


**Supplementary Figure1. The forest plots of subgroups for risk of hospitalization stratified by age (Omicron *vs.*Delta)**

**
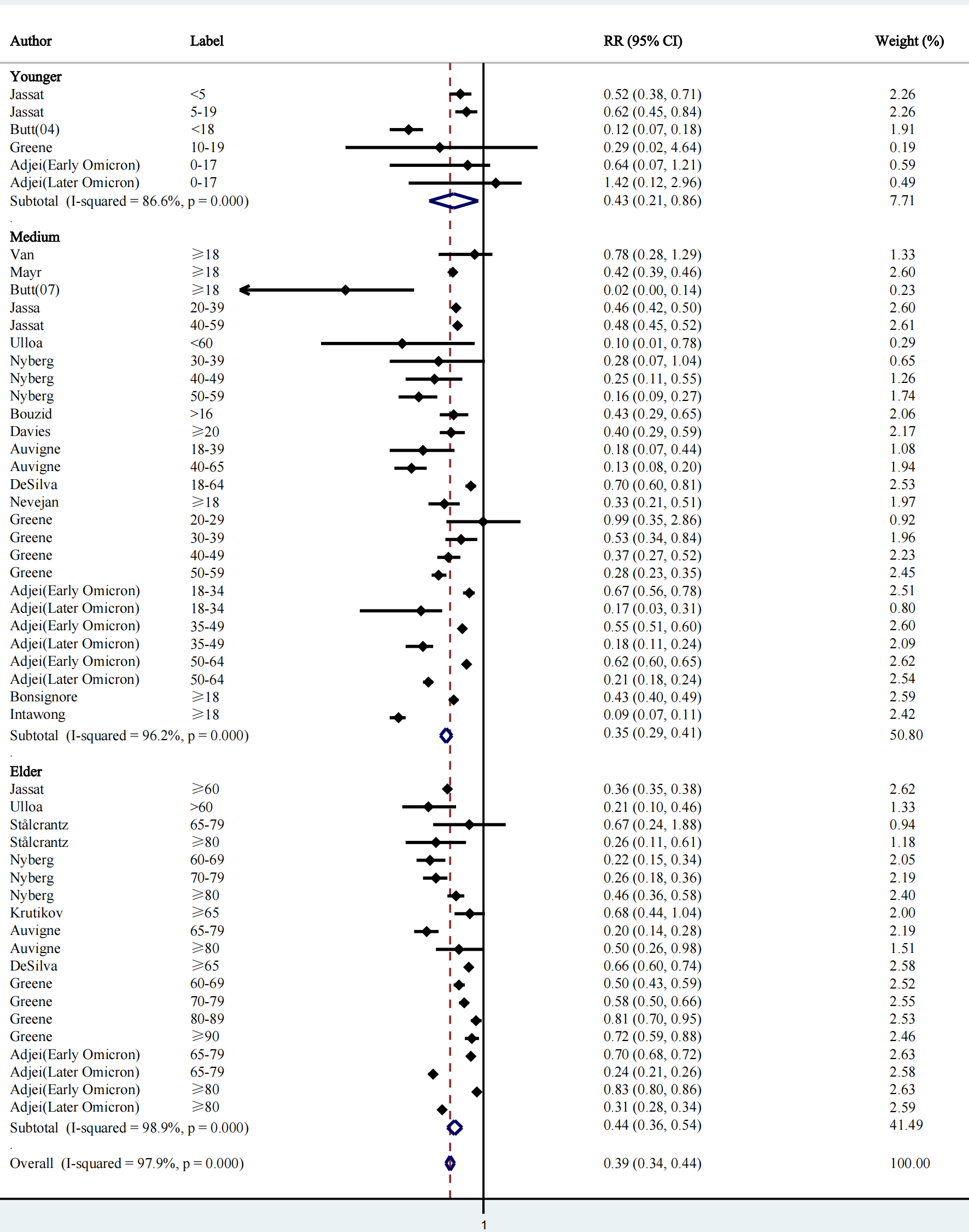
**

**Supplementary Figure2. The forest plots of subgroups for risk of death stratified by age (Omicron *vs.*Delta)**


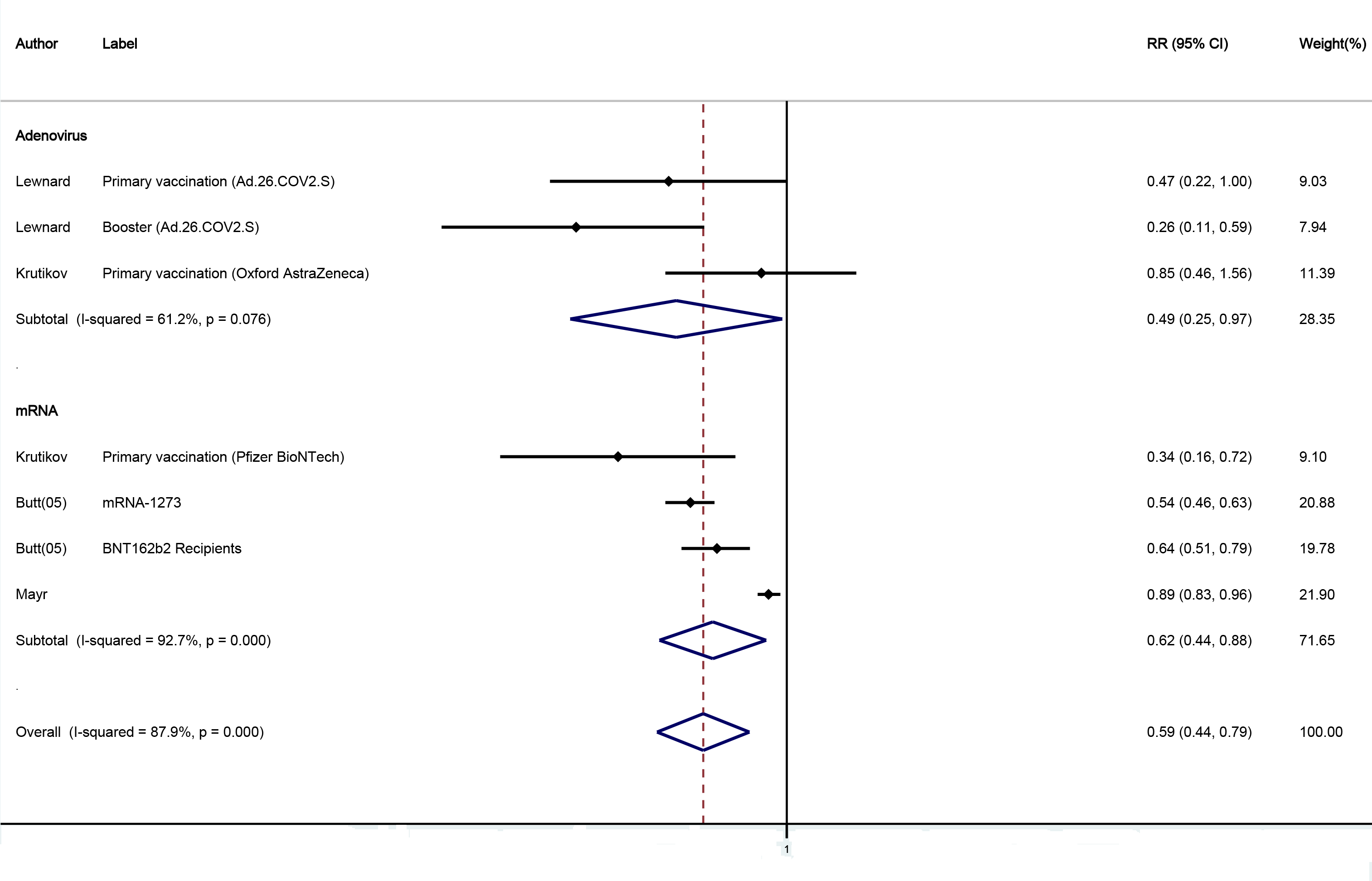


**Supplementary Figure 3. The forest plots of subgroups for risk of hospitalization stratified by type of vaccine (Omicron *vs.*Delta)**

**
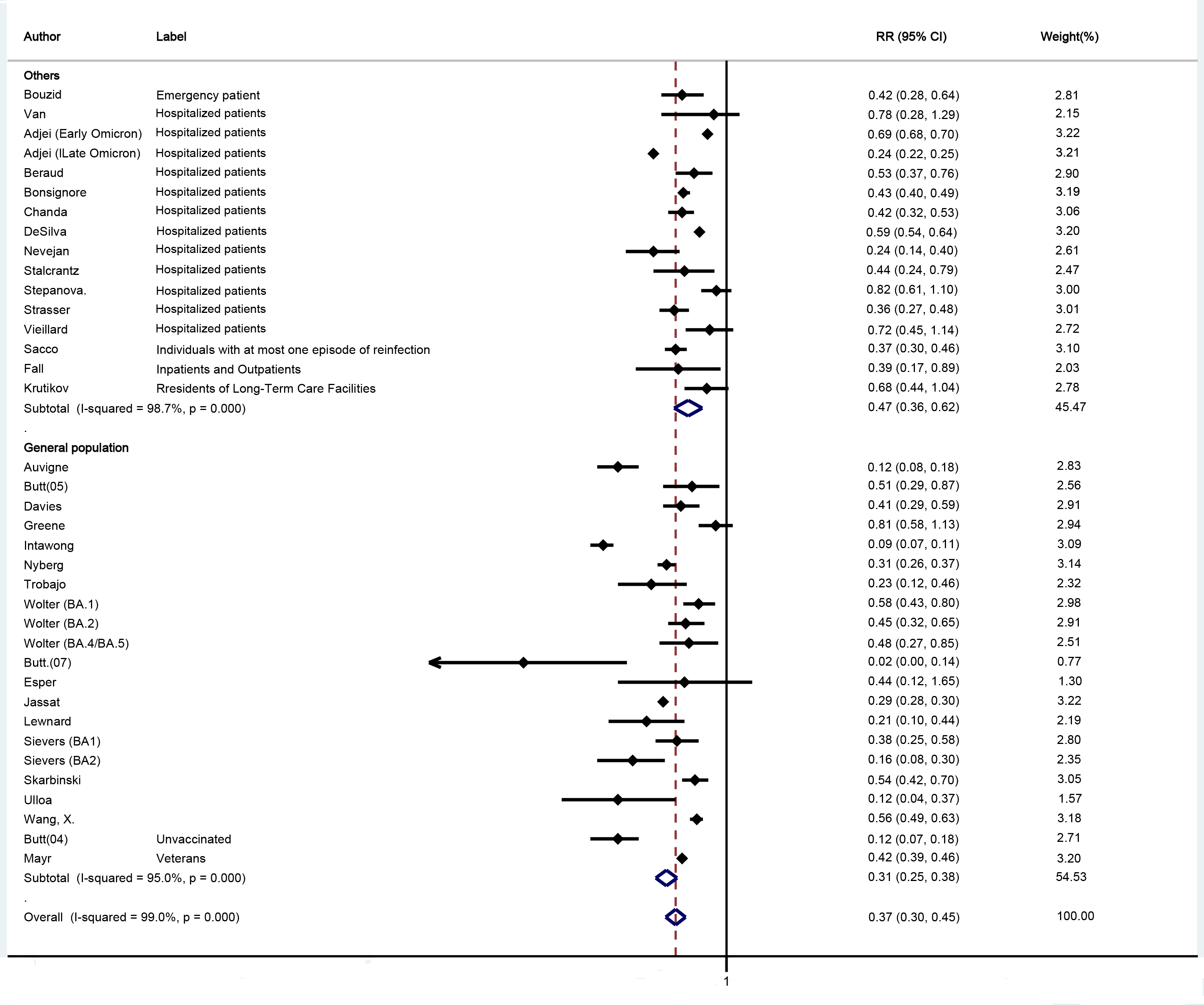
**

**Supplementary Figure 4. The forest plots of subgroups for risk of death stratified by patient source (Omicron *vs.*Delta)**

**
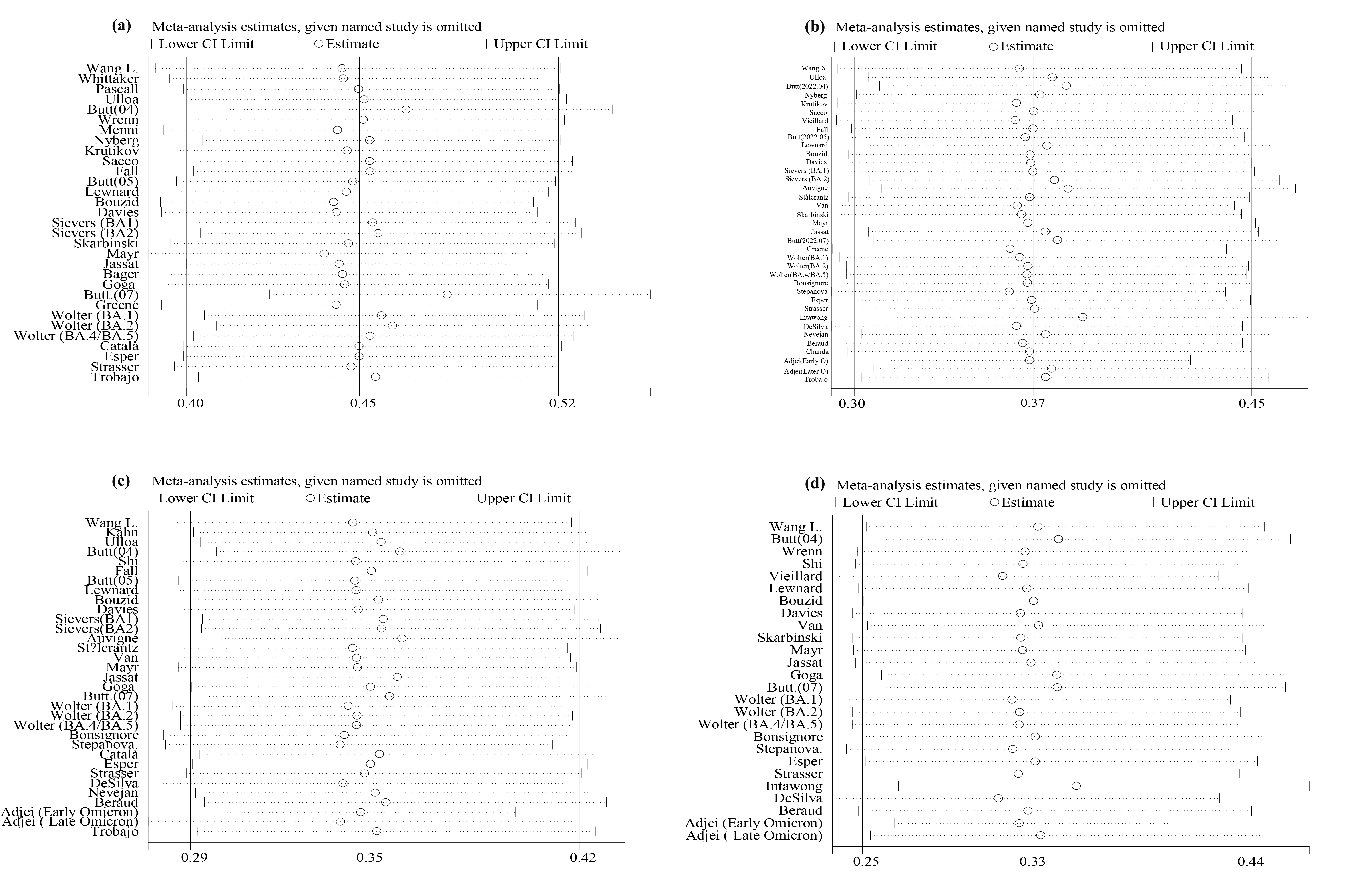
**

**Supplementary Figure 5. Sensitivity analysis for risk of (a) hospitalization. (b) death. (c) ICU admission.(d) mechanical ventilation.**

**
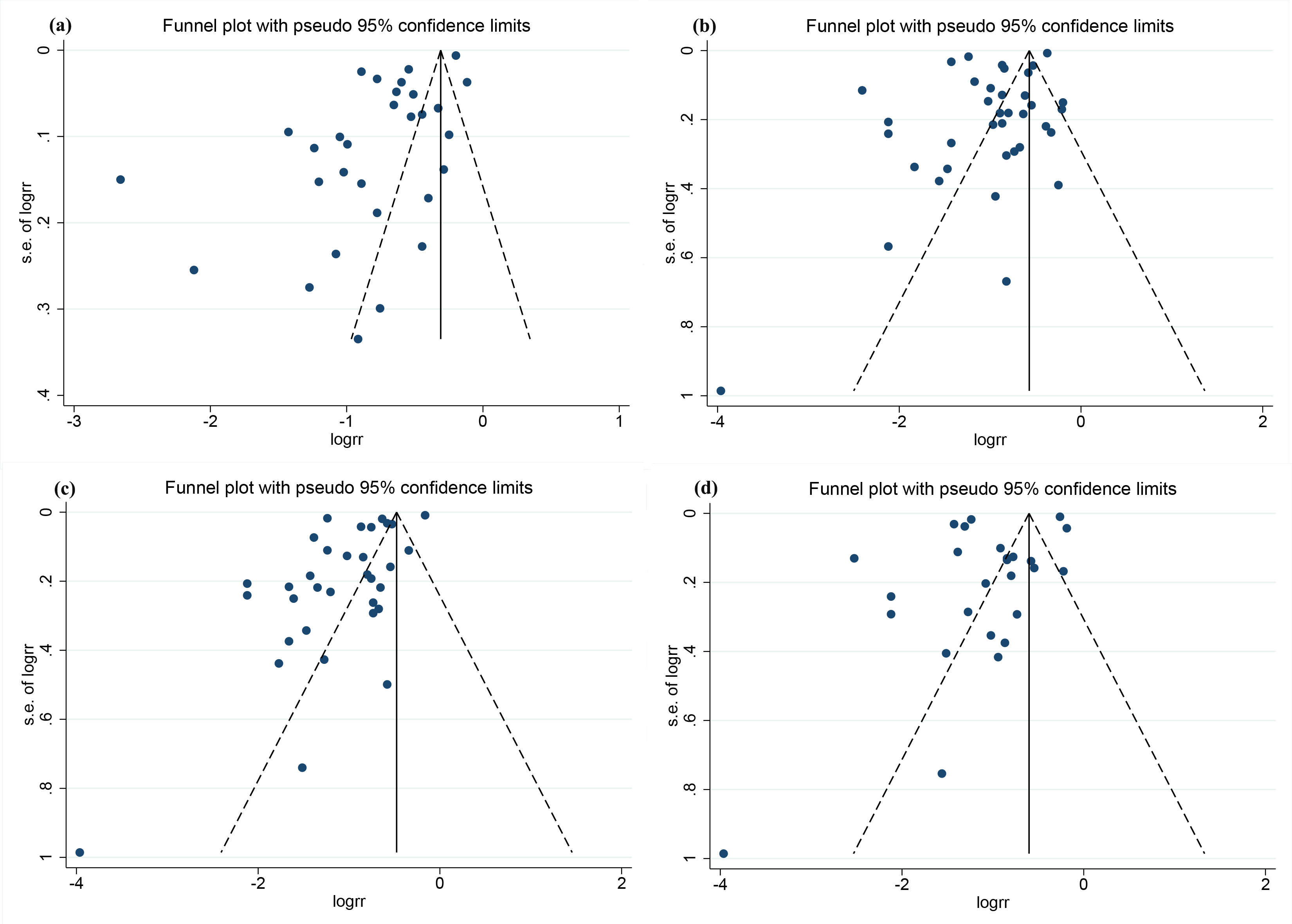
**

**Supplementary Figure 6. Funnel plot for risk of (a) hospitalization. (b) death. (c) ICU admission.(d) mechanical ventilation.**
